# Integrating humans into pace-of-life studies: The Big Five personality traits and metabolic rate in young adults

**DOI:** 10.1371/journal.pone.0248876

**Published:** 2021-04-06

**Authors:** Patrick Bergeron, Ariane Pagé, Maxime Trempe

**Affiliations:** Bishop’s University, Sherbrooke, Quebec, Canada; University of Minnesota, UNITED STATES

## Abstract

The pace-of-life syndrome (POLS) predicts that personality and metabolism should be correlated if they function as an integrated unit along a slow-fast continuum. Over the last decade, this conceptual framework has been tested in several empirical studies over a wide array of non-human animal taxa, across multiple personality traits and using standardized measures of metabolism. However, studies associating metabolic rate and personality in humans have been surprisingly scarce. Here, we tested whether there was covariation among personality scores, measured using the Big Five Inventory test, resting metabolic rate (RMR) and preferred walking speed (PWS) in a cohort of young human adults aged between 18 and 27 years old. We found a significant, negative relationship between RMR and Extraversion; less extraverted individuals had a 30% higher RMR than the most extraverted ones. No other personality traits correlated with RMR and none correlated with PWS. The negative correlation between Extraversion and RMR may suggest an allocation energy trade-off between personality and basal metabolism. Our results yielded equivocal support for the POLS and emphasized the need for more research on human to test the generality of this conceptual framework and further assess its validity.

## Introduction

Recent theories have suggested that physiological, personality and life-history traits must be in phase with one another to provide optimal organismal functioning [1–4]. In evolutionary biology, the pace-of-life syndrome (POLS) hypothesis is evoked to explain the maintenance of genetic variation for traits that mediate the trade-off between current and future reproduction. These traits are expected to align along a slow-fast pace-of-life continuum such that “fast”/short-lived individuals would have, for example, a high metabolism and high activity to sustain a fast reproductive rate, while “slow”/long lived individuals would have the opposite [3, 5, 6]. However, empirical support for the consistent covariance between personality and metabolic traits predicted by the POLS remains equivocal when assessed using non-human animal species [7, 8]. For example, despite the expectation that individuals scoring high on activity, aggressiveness and boldness scales should have a higher metabolic rate than individuals with lower scores, many studies have found no relationships between these traits (reviewed in [8]). These results could be due, in part, to the relatively high discrepancy between the methodologies used to assess personality traits across different species [7, 9, 10]. Because humans are among the most well-studied species, integrating covariations between personality and metabolism into the POLS could help to further test this conceptual framework and assess its validity [11].

Human subjects offer an interesting avenue to study personality and its relationship with POLS because standardized and well-accepted questionnaires are readily available [12]. For example, the classic Big Five Inventory test [13], which characterizes personality along five dimensions (Openness, Conscientiousness, Extraversion, Agreeableness and Neuroticism), has been extensively used to investigate how human personality correlates with individual states and behaviors (e.g., [13–15]). Several studies have shown that personality traits do correlate with life-histories, thus supporting the idea that the POLS could apply to humans. For instance, Jokela and colleagues [16] conducted a large-scale study of the relationship between human personality and mortality from all causes and found that low conscientiousness was related to a higher risk of mortality. Additionally, other studies have shown that extraversion was positively linked with the number of sexual partners, family size, and the likelihood of being involved in life-threatening accidents [17, 18]. Based on these results, one could argue that high conscientiousness and high extraversion may be associated with a fast pace of life [19, 20].

There are also indications in humans that metabolic rate may be attuned to personality. In a seminal work on the subject, a relationship was reported between the Psychopathic Deviate scale of the Minnesota Multiphasic Personality Inventory and the participants’ blood pressure (young police recruits; [21]). Recently, Lehmann et al. [11] studied the relationship between maturation status and risk-taking behavior in a sample of more than a thousand German adolescents and showed that fast-maturing adolescents had higher blood pressure and expressed more health-related risk-taking behaviors compared to slow-maturing ones. Notably, because humans have varying levels of resting metabolic rates (RMRs) based on their body composition and metabolism [22], the POLS would predict a relationship between RMR and personality. To our knowledge, this relationship has only been investigated by Terracciano and colleagues [23], who reported no relationship between personality and RMR in a cohort of participants having a mean age of 67 years old. Since RMR has been repeatedly shown to decrease with age [22], it remains unknown whether such a relationship exists in younger adults. The authors however reported that energy expenditure at peak walking pace was positively related to Extraversion, Openness and Conscientiousness and was negatively related to Neuroticism, thus reinforcing the idea that personality may be linked to metabolism.

Here, we tested whether there was covariation between personality traits indexed by the Big Five Inventory test and metabolism in a cohort of human adults aged between 18 and 27 years old. The RMR and preferred walking speed (PWS) of all participants were used as indicators of the energy requirements for basal physiological functioning and energy expenditure during locomotion, respectively [24]. The POLS predicts that personalities associated with energy demanding behaviors should correlate positively with energy expenditure [3]. One could speculate that Extraversion, with facets such as Activity Level and Excitement Seeking, as well as Neuroticism, with facets like Depression and Vulnerability, may correlate more strongly with energy metabolism compared to Openness, Agreeableness and Conscientiousness, for which the underlying facets relate predominantly to thoughts and affective states [25]. Our goal was thus to characterize the relationship between personality and metabolism and assess whether the patterns observed, if any, are indicative of a POLS.

## Methods

### Overview

Data were recorded in a single visit during which participants were asked to complete the Big Five Inventory test and to undergo RMR and PWS tests. Forty participants (22 male, 18 female, *M*_*age*_ *=* 21.0, *SD*_*age*_ = 2.2) participated in the experiment. The participants were recruited on the Bishop’s University campus, Canada, via recruitment posters and social media advertisements. The protocol and procedures were reviewed and approved by the Ethics Research Board of Bishop’s University, and the methods were in accordance with the relevant guidelines and regulations. The participants provided written consent for the study and received $10 compensation to participate in this project.

### Personality and physiological assessment

After signing a written statement of informed consent, the participants were asked to complete a demographic questionnaire. This questionnaire allowed to obtain information about potential covariates such as the participants’ age, gender, exercise habits (i.e., usual exercising time per week), time since their last meal and height. We also measured their body mass to report RMR as O_2_ ml/kg/min. The participants were then seated and asked to complete the 44-item Big Five Inventory test [13]. In brief, the participants had to indicate their level of agreement with each of the 44 statements using a 5-point Likert scale. Using the questionnaire’s scoring grid, the answers were converted into an average score for each personality dimension.

The participants were then asked to remain seated and to refrain from moving for 15 minutes. During this time, the experimenter placed on the participant’s face the mask used to measure the RMR (A-GAK-201-S) and calibrated the Iworx TA (GA-200, Dover, NH) gas analyzer using a 3L calibration syringe (Roxon, Canada) and a gas mixture of known concentrations (O_2_: 12%, CO_2_: 5%). Following the 15-minute rest interval, oxygen uptake was measured continuously for 8 minutes using LabScribe (v3.6) software. Resting rate oxygen consumption (VO_2_) was calculated using the consumption value over the last 5 minutes of the test and reported relative to body weight and time (O_2_ ml/kg/min).

To measure the PWS, the participants were invited to walk on a treadmill for 10 minutes at the speed of their choice. The participants were naïve to the purpose of this test; they were simply told by the experimenter to walk at a comfortable speed (i.e., their usual walking speed) and that they could adjust by themselves the treadmill speed. They could make as many adjustments to the treadmill speed as they wanted, and the speed chosen during the last five minutes of the test was considered their preferred walking speed.

### Statistical analyses

**Fig 1:**
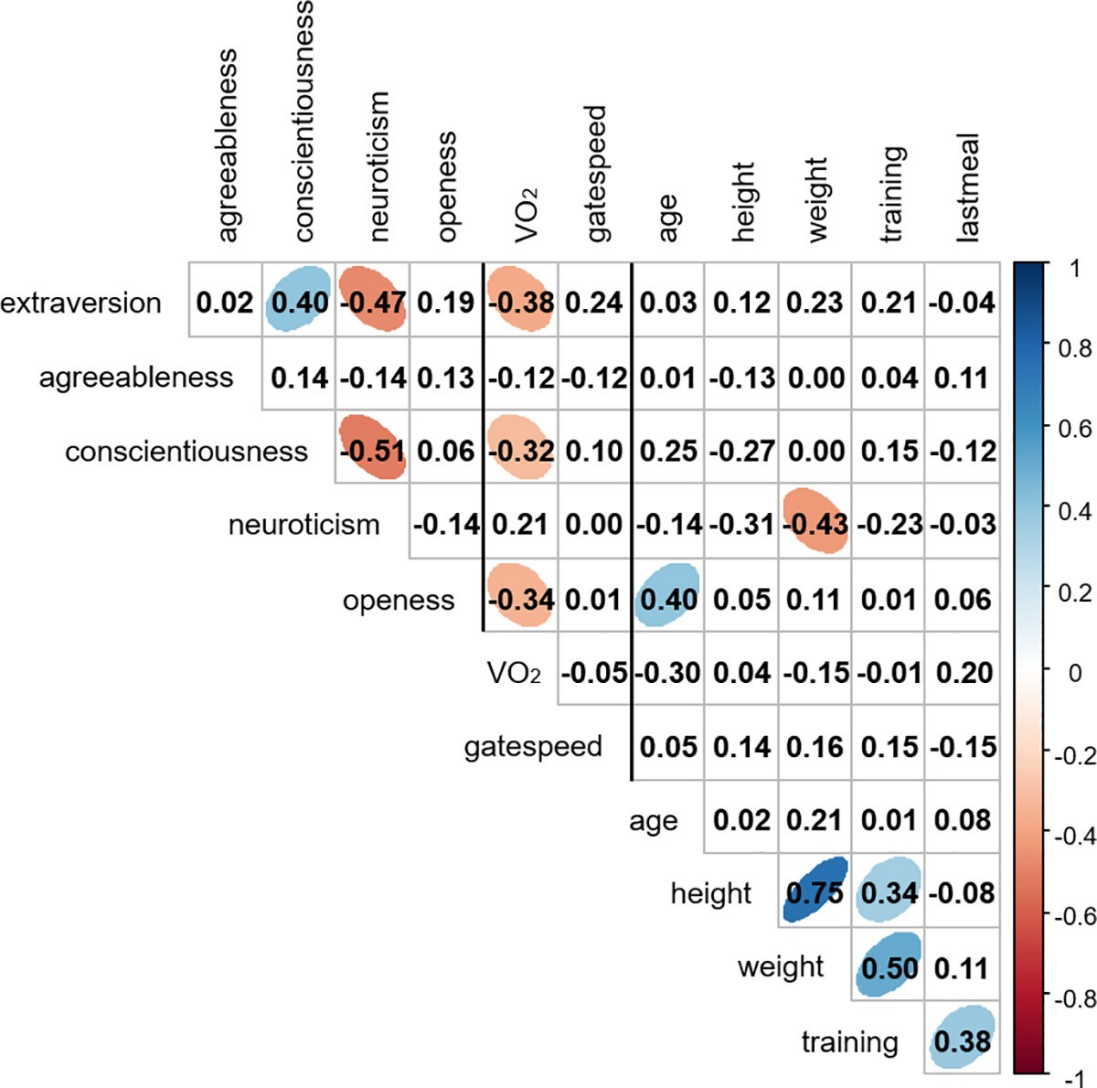
Bivariate correlation matrix of all continuous variables (*n* = 40 participants). Pearson’s correlation coefficients are shown. Ellipses highlight significant correlations. The color scale represents the direction and strength of the correlation coefficient.

RMR and PWS were each tested using linear models and a Gaussian distribution to test their relationships with the five personality scores. For both models, we used a two-step approach in which we first investigated the effect of the potential covariates Age, Gender, Exercise habit (number of minutes per week), and Time since the last meal (in minutes) on RMR and PWS. Because of its relationship with stride length, height was also included in the PWS model. In the second step, we only included the significant covariates (age for RMR and none for PWS, see results) in the models containing also the five personality scores as fixed effects. Bivariate correlations of all continuous variables are presented in Fig 1.

We conducted Shapiro-Wilk and Levene tests to confirm that our data were normally distributed and had homogeneity of variance. We computed all statistical analyses using the programming environment R (v.3.6.1). We used a stepwise backward model selection approach, sequentially removing the least non-significant term from the model based on its *p*-value (α = 0.05). All results are reported as slope ± SE.

## Results

**Fig 2:**
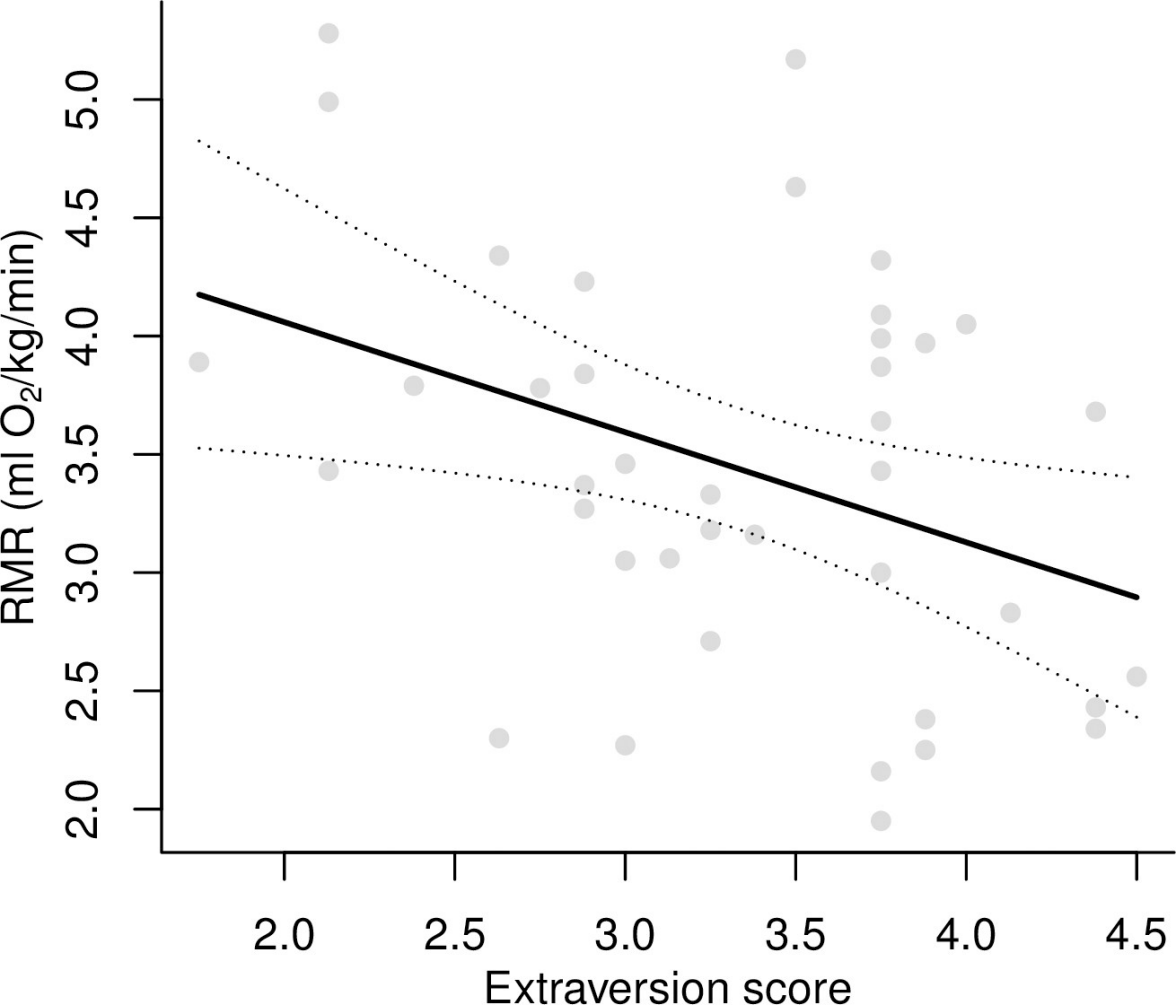
Relation between Extraversion score and Resting Metabolic Rate (RMR) while accounting for the effect of age. The gray dots represent the raw data, while the solid and dotted lines illustrate the model’s prediction along with the 95% CI, respectively.

Variables of interest and their potential covariates are summarized in Table 1 and Fig 1. There were no relationships of RMR with Gender (*p* = 0.22), Exercise habit (*p* = 0.23) and Time since the last meal (*p* = 0.14). However, there was a marginally non-significant negative relationship between RMR and Age (-0.12 ± 0.06, *t* = 1.91, *p* = 0.06); Age was therefore kept in the main analysis with personality. We found a strong, significantly negative relationship between RMR and Extraversion (Table 2A, Fig 2). RMR decreased by 30% (from 4.16 to 2.91 O_2_ ml/kg/min) over the range of observed Extraversion scores (1.75 to 4.5). Age tended to be negatively related to RMR in this model, but none of the other personality scores were (Table 2A).

**Table 1:** Descriptive statistics of the variables of interest on 40 young adults (*n* = 22 male, 18 female).

Variables	Mean	*SD*
Personality		
Agreeableness	3.9	0.4
Conscientiousness	3.4	0.5
Extraversion	3.3	0.7
Neuroticism	2.7	0.8
Openness	3.5	0.5
Metabolism		
RMR (O_2_ ml/kg/min)	3.4	0.9
PWS (km/h)	3.2	0.6
Covariates		
Age	21.0	2.2
Body weight (kg)	74.7	18.5
Height (cm)	173.5	10.7
Exercise habit (min/week)	400.5	311.0
Time since last meal (min)	162.4	105.7

RMR and PWS stand for resting metabolic rate and preferred walking speed, respectively. Personality scores range from 1 to 5, with 5 indicating a high score on the trait.

**Table 2:** a) Relationship between the personality scores and resting metabolic rate (RMR) while accounting for the age of the participants (*n* = 40). b) Relationship between personality scores and preferred walking speed (PWS). Statistics are provided when the terms were removed from the model during the backward selection process.

	Estimate	SE	*t*-value	*p*-value
a) RMR				
Age	-0.11	0.06	1.96	0.06
Extraversion	-0.45	0.18	2.53	0.02
Agreeableness	-0.13	0.29	0.46	0.65
Conscientiousness	-0.26	0.27	0.97	0.34
Neuroticism	-0.08	0.19	0.43	0.67
Openness	-0.35	0.29	1.22	0.23
b) PWS				
Extraversion	0.20	0.13	1.53	0.13
Agreeableness	-0.17	0.20	0.82	0.42
Conscientiousness	0.09	0.21	0.43	0.67
Neuroticism	0.09	0.13	0.68	0.50
Openness	-0.01	0.20	0.08	0.94

Bivariate correlations suggested that Extraversion, Conscientiousness and Openness were correlated with RMR (Fig 1). However, Conscientiousness was not related to RMR when Extraversion was included in the model, and Openness was not related to RMR when age, although marginally non-significant, was included in the model (Table 2A). To further investigate the relationship between RMR and Openness, we ran the analysis with the five personality scores without age. The final model was the same as the model with age with respect to Extraversion (-0.47 ± 0.19, *t* = 2.5, *p* = 0.02), while Openness tended to also be negatively related to RMR, but the effect was not significant (-0.50 ± 0.27, *t* = 1.89, *p* = 0.07).

Regarding PWS, there was no relationship with any of the covariates: Age (*p* = 0.67), Gender (*p* = 0.75), Exercise habit (*p* = 0.35), Time since last meal (*p* = 0.17) and Height (*p* = 0.97). There was also no significant relationship between PWS and any of the five personality scores (Table 2B).

Internal consistency measured with Cronbach’s alpha coefficients were relatively high for Extraversion (0.84) and Neuroticism (0.86), while they were lower for Conscientiousness (0.72), Openness (0.67) and Agreeableness (0.64).

## Discussion

Our goal was to examine the correlations between the Big Five personality traits and metabolism in young adults. This research was inspired by the vast literature on the POLS using non-human animals, with the hope that standardized personality traits could contribute to reducing the measurement noise around this construct [7]. Our main result was that Extraversion correlated negatively with RMR. All other traits showed no correlations with RMR, and none of the traits was correlated with PWS.

While never reported before, the significant correlation between Extraversion and RMR is coherent with previous personality work. According to DeYoung’s cybernetic model [14], Extraversion and Openness share a common variance and can be regrouped under the metatrait Plasticity, which indicates one’s “tendency toward behavioral exploration, using motor output to pursue potentially rewarding possibilities […]”. In addition, when assessed using the 44-item Big Five Inventory test (as in the present study), Extraversion included several questions pertaining to the facet Activity [26]. This language is strikingly similar to that used in animal studies, in which “activity” and “exploration” scores are personality traits that have been shown to covary with metabolism [3]. In fact, when used in animal studies, “personality” cannot be dissociated from behavior since observing the animal’s behavior is the only way in which researchers can infer the animal’s personality [27]. It therefore may not come as a surprise if, in our dataset, the only significant correlation between RMR and personality was obtained with Extraversion, the trait highest in visibility and the one that can be most accurately assessed by other raters [28]. Thus, our results suggest that the components of personality linked to behavior and movements are attuned to the body metabolism.

The negative correlation between RMR and Extraversion, however, adds to a growing number of reports failing to support the POLS [7]. More specifically, the POLS would predict that personalities associated with energy-demanding behaviors should positively correlate with basal energy expenditure and not the opposite [3]. In contrast, Careau et al. [29] suggested that a negative correlation between personality and metabolism could be observed if “fast” personalities are maintained via an energetic allocation trade-off with metabolism. In other words, individuals could be able to sustain energetically demanding personalities (or the behaviors associated with these personalities) by spending less energy at rest. Such a trade-off has been observed between metabolic rate and boldness in fall field crickets (*Gryllus pennsylvanicus*) [30] and with activity in mosquito fish (*Gambusia holbrooki*) [31]. In humans, there have been suggestions that total daily energy expenditure is bounded to a fixed level such that an increase in daily energy expenditure (e.g., by engaging in physical activity) is associated with a corresponding decrease in basal energy expenditure [32]. Our results support the energy trade-off model and suggest that the energy cost associated with Extraversion (and, indirectly, the metatrait Plasticity) are compensated for by a decrease in basal metabolic rate. This conclusion is also coherent with Terracciano et al.’s observation that individuals who scored high on Extraversion saved energy by increasing their walking efficiency [23]. Whether individuals can energetically “afford” to be extraverted because of their metabolism or whether metabolism adapts to sustain the energetically demanding behaviors of some personality traits remains open for investigation.

The finding that Agreeableness, Conscientiousness, Neuroticism and, to a certain extent, Openness were not correlated with RMR can be interpreted in different ways. First, because these traits mainly encompass facets related to internal thoughts and affective states, it is possible that they vary independently from metabolism. However, we cannot exclude the possibility that a relationship does exist, but we were unable to capture it. As demonstrated before, these traits are higher in evaluativeness compared to Extraversion, indicating that social norms and values can influence how one responds to their associated questions [28]. Since our experiment was conducted on a small and intimate university campus, it is possible our participants’ responses were, intentionally or unintentionally, biased. This possibility could explain the rather low Cronbach’s alpha that we reported for these traits. In our context, an other-rater procedure may have led to a more accurate evaluation. Alternatively, our analysis may have been underpowered to detect a relationship of this size, making a larger sample size more desirable in future studies. Considered together, our results demonstrate the importance of further exploring the energetic costs of personality traits and their possible variations over the full life span.

The failure to observe a relationship between personality and PWS on a treadmill is difficult to interpret because the participants’ familiarity with this equipment was not homogenous in our sample. Future studies may want to utilize a more ecological measurement, such as the average walking speed over a 24-hour period assessed using a GPS or smartphone. In addition, the correlative nature of this research, using a single point measurement per subject and small sample size, requires caution in inferring causality since our approach does not allow for distinguishing among- and inter-individual contributions to the observed phenotypic correlation [33]. Additionally, we cannot exclude the possibility that a third, unmeasured trait affects the observed phenotypic correlation. For instance, ethnicity and time since last exercise could have been relevant control variables [22]. Nevertheless, our results raise important questions about the expected relationship between personality and metabolism within the POLS conceptual framework and highlight the importance of better understanding models of energy allocation.

## References

[1] Ricklefs RE, Wikelski M. The physiology/life-history nexus. Trends Ecol Evol. 2002;17: 462–468.

[2] Sih A, Bell A, Johnson JC. Behavioral syndromes: An ecological and evolutionary overview. Trends Ecol Evol. 2004;19: 372–378. 10.1016/j.tree.2004.04.009 16701288

[3] Réale D, Garant D, Humphries MM, Bergeron P, Careau V, Montiglio P-O. Personality and the emergence of the pace-of-life syndrome concept at the population level. Phil Trans R Soc B Biol Sci. 2010;365: 4051–4063. 10.1098/rstb.2010.0208 21078657 PMC2992747

[4] Dammhahn M, Dingemanse NJ, Niemelä PT, Réale D. Pace-of-life syndromes: a framework for the adaptive integration of behaviour, physiology and life history. Behav Ecol Sociobiol. 2018;72: 62.

[5] Stamps JA. Growth-mortality tradeoffs and “personality traits” in animals. Ecol Lett. 2007;10: 355–363. 10.1111/j.1461-0248.2007.01034.x 17498134

[6] Biro PA, Stamps JA. Are animal personality traits linked to life-history productivity? Trends Ecol Evol. 2008;23: 361–368. 10.1016/j.tree.2008.04.003 18501468

[7] Royauté R, Berdal MA, Garrison CR, Dochtermann NA. Paceless life? A meta-analysis of the pace-of-life syndrome hypothesis. Behav Ecol Sociobiol. 2018;72: 64.

[8] Careau V, Garland T. Performance, ersonality, and energetics: correlation, causation, and mechanism. Physiol Biochem Zool. 2012;85: 543–571. 10.1086/666970 23099454

[9] Montiglio P, Dammhahn M, Dubuc-Messier G, Réale D. The pace-of-life syndrome revisited: the role of ecological conditions and natural history on the slow-fast continuum. Behav Ecol Sociobiol. 2018;72: 116.

[10] Smith BR, Blumstein DT. Fitness consequences of personality: A meta-analysis. Behav Ecol. 2008;19: 448–455.

[11] Lehmann A, Eccard JA, Scheffler C, Kurvers R, Dammhahn M. Under pressure: human adolescents express a pace-of-life syndrome. Behav Ecol Sociobiol. 2018;72: 57.

[12] McCrae RR, Costa PT. Personality trait structure as a human universal. Am Psychol. 1997;52: 509–516. 10.1037//0003-066x.52.5.509 9145021

[13] John OP, Srivastava S. Handbook of personality: theory and research. Pervin LA, John O., editors. New York: Guilford Press; 1999.

[14] DeYoung CG. Cybernetic Big Five Theory. J Res Pers. 2015;56: 33–58.

[15] Digman JM. Personality structure: emergence of the five-factor model. Annu Rev Psychol. 1990;41: 417–440.

[16] Jokela M, Batty GD, Nyberg ST, Virtanen M, Nabi H, Singh-manoux A, et al. Personality and all-cause mortality: individual-participant meta-analysis of 3,947 deaths in 76,150 adults. Am J Epidemiol. 2013;178: 667–675. 10.1093/aje/kwt170 23911610 PMC3755650

[17] Nettle D. The evolution of personality variation in humans and other animals. Am Psychol. 2006;61: 622–631. 10.1037/0003-066X.61.6.622 16953749

[18] Alvergne A, Jokela M, Lummaa V, Nisbett RE, Alvergnea A, Lummaa V. Personality and reproductive success in a high-fertility human population. Proc Natl Acad Sci. 2010;107: 11745–11750. 10.1073/pnas.1001752107 20538974 PMC2900694

[19] Lukaszewski AW, Rueden CR Von. The extraversion continuum in evolutionary perspective: a review of recent theory and evidence. Pers Individ Dif. 2015;77: 186–192.

[20] Carter NT, Guan L, Maples JL, Williamson RL, Miller JD. The downsides of extreme conscientiousness for psychological well-being: the role of obsessive compulsive tendencies. J Pers. 2016;84: 510–522. 10.1111/jopy.12177 25858019

[21] Hogan J. Personality correlates of physical fitness. J Pers Soc Psychol. 1989;56: 284–288. 10.1037//0022-3514.56.2.284 2926630

[22] McMurray RG, Soares J, Caspersen CJ, McCurdy T. Examining variations of resting metabolic rate of adults: a public health perspective. Med Sci Sports Exerc. 2014;46: 1352–1358. 10.1249/MSS.0000000000000232 24300125 PMC4535334

[23] Terracciano A, Schrack JA, Sutin AR, Chan W, Simonsick EM, Ferrucci L. Personality, metabolic rate and aerobic capacity. PLoS One. 2013;8. 10.1371/journal.pone.0054746 PMC3556088 23372763

[24] Brooks G, Fahey T, Baldwin K. Exercise physiology: human bioenergetics and its applications: human bioenergetics and its applications. McGraw-Hill Education; 2004.

[25] Johnson JA. Measuring thirty facets of the Five Factor Model with a 120-item public domain inventory: development of the IPIP-NEO-120. J Res Pers. 2014;51: 78–89.

[26] Soto CJ, John OP. Ten facet scales for the Big Five Inventory: convergence with NEO PI-R facets, self-peer agreement, and discriminant validity. J Res Pers. 2009;43: 84–90.

[27] Gosling SD. From mice to men: what can we learn about personality from animal research? Psychol Bull. 2001;127: 45–86. 10.1037/0033-2909.127.1.45 11271756

[28] Connelly BS, Ones DS. An other perspective on personality: meta-analytic Integration of Observers’ Accuracy and Predictive Validity. Psychol Bull. 2010;136: 1092–1122. 10.1037/a0021212 21038940

[29] Careau V, Thomas D, Humphries MM, Réale D. Energy metabolism and animal personality. Oikos. 2008;117: 641–653.

[30] Careau V, Beauchamp PP, Bouchard S, Morand-Ferron J. Energy metabolism and personality in wild-caught fall field crickets. Physiol Behav. 2019;199: 173–181. 10.1016/j.physbeh.2018.11.023 30465808

[31] Biro PA, Thomas F, Ujvari B, Adriaenssens B, Beckmann C. Spontaneous activity rates and resting metabolism: support for the allocation model of energy management at the among‐individual level. Ethology. 2019; 1–8.

[32] Pontzer H, Durazo-Arvizu R, Dugas LR, Plange-Rhule J, Bovet P, Forrester TE, et al. Constrained total energy expenditure and metabolic adaptation to physical activity in adult humans. Curr Biol. 2016;26: 410–417. 10.1016/j.cub.2015.12.046 26832439 PMC4803033

[33] Niemelä PT, Dingemanse NJ. On the usage of single measurements in behavioural ecology research on individual differences. Anim Behav. 2018;145: 99–105.

